# Regulation of murine natural killer cell commitment

**DOI:** 10.3389/fimmu.2013.00014

**Published:** 2013-01-30

**Authors:** Nicholas D. Huntington, Stephen L. Nutt, Sebastian Carotta

**Affiliations:** ^1^Molecular Immunology Division, Walter and Eliza Hall Institute of Medical ResearchParkville, VIC, Australia; ^2^Department of Medical Biology, The University of MelbourneParkville, VIC, Australia

**Keywords:** innate lymphocytes, natural killer cells, development, pre-pro NK cells, transcription factors, differentiation, ID2, NFIL3

## Abstract

Natural killer (NK) cells can derive from the same precursors as B and T cells, however, to achieve lineage specificity, several transcription factors need to be activated or annulled. While a few important transcription factors have been identified for NK genesis the mechanisms of how this is achieved is far from resolved. Adding to the complexity of this, NK cells are found and potentially develop in diverse locations *in vivo* and it remains to be addressed if a common NK cell precursor seeds diverse niches and how transcription factors may differentially regulate NK cell commitment in distinct microenvironments. Here we will summarize some recent findings in NK cell commitment and discuss how a NK cell transcriptional network might be organized, while addressing some misconceptions and anomalies along the way.

## GENERAL INTRODUCTION OF NK CELLS

Natural killer (NK) cell research is experiencing somewhat of a revolution. Gone are the days when NK cells could be described as a homogenous innate lymphoid cells (ILC) population producing inflammatory cytokines and cytotoxic granules when encountering cells with missing/altered self-MHC-I expression. Now NK cells can be viewed as a “jack-of-all-trades”; a diverse lineage implicated in innate and adaptive immunity where specification to pathogen response is shaped by parameters such as maturation state, anatomical location, interaction with self-MHC-I, history of inflammation or pathogen exposure, receptor expression, growth factor responsiveness, and survival status. Despite these advances in our understanding of effector and regulatory functions of NK cells, many key questions surrounding their origin and development remain.

Natural killer cells are ILCs and like B and T cells, NK cells undergo an ordered and rather linear maturation process. However, unlike B and T cells that take longer to develop and become fully capable of performing their effector functions, mature NK cells emerge early and are rapidly activated upon detection of pathogens (Huntington et al., 2007b). So what is unique about the genetic programing of the NK cell lineage that sets them apart from other lymphocytes? Of course, we must generalize a little when discussing NK cells, as this population appears profoundly more diverse than we first envisaged (Huntington et al., 2007b). NK cells represent a considerable fraction of mononuclear cells in human peripheral blood and while this fraction is somewhat less in mice, NK cells can be found in all secondary lymphoid organs and associated lymphoid tissues in both species (Huntington et al., 2007b). The origin of conventional NK (cNK) cells is the bone marrow (Haller et al., 1977). At this site, lymphoid progenitors capable of differentiating into B, T, and NK cells, either via environmental cues or stochastic gene expression, are committed toward one of these lineages (Kondo et al., 1997). This process involves large-scale genetic changes within the committing cells, ultimately activating genes of one lineage while repressing genes of other lineages. B and T lymphoid commitment is better understood than that of NK cells and a set of key genetic regulators of B and T cells fate have been identified and their expression consigns progeny to that lineage (Warren and Rothenberg, 2003). In the case of B cells, Pax5 represses non-B cell genes while activating B cell-specific genes while E2A/Notch1/TCF7/bcl11b perform similar roles in T cells (Nutt et al., 2001; Warren and Rothenberg, 2003). However, a “master regulator” of the NK cell lineage remains elusive. Here we discuss the recent advances that have been made in the field of transcriptional regulation of NK cell lineage commitment.

### NK CELL DEVELOPMENT

Natural killer cells can be grouped based on their organ of development: bone marrow-derived or cNK cells, thymic NK cells, fetal liver NK cells, and potentially intestinal NK cells.

### cNK CELL DEVELOPMENT

**FIGURE 1:**
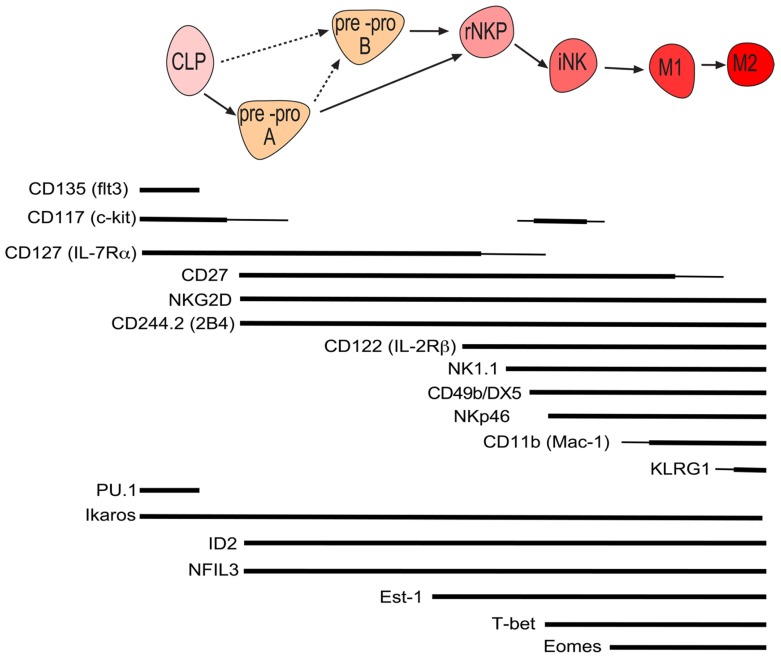
**Schematic of murine conventional NK cell development with the cell surface phenotype and required genes at each stage of development**. Line width is proportional to expression level.

Conventional NK cells constitute the major and best characterized population of NK cells and are formed from lymphoid progenitors in the bone marrow (**Figure 1**). To search for the earliest committed NK cell progenitor (NKP), we recently made use of an inhibitor of DNA binding 2 (ID2)-GFP reporter mouse where GFP was expressed in all cNK cells under the control of the ID2 promoter (Carotta et al., 2011; Jackson et al., 2011). Analysis of the bone marrow revealed that common lymphoid progenitors (CLPs) were GFP^-^, however, a small population of GFP^+^ cells was detected in the FLT3^-^ CLP fraction. These GFP^+^ cells were further characterized Lin-ID2^+^Sca1^+^CD127^+^CD117^low^CD135^-^CD122^-^ and efficiently differentiated into NK cells *in vitro* while having lost T and B cell potential (Carotta et al., 2011). A similar population was identified amongst the Lin-Sca1^+^CD117^-^ fraction and while we termed the CD117^low^ population pre-pro A and the CD117^-^ population pre-pro B NK cells, the relationship between the two subsets remains is unresolved. Prior to the generation of ID2-GFP mice, the earliest committed NKP was identified through the expression of the IL-2 receptor β chain and the absence of lineage markers including the pan-NK cell markers NK1.1 and CD49b (DX5; Rosmaraki et al., 2001). However, this population was heterogeneous with only a minority of cells being NK cell committed. We purified the NKP within the lin^-^CD122^+^NK1.1^-^CD49b^-^ population using ID2-GFP mice and found that NKP cells could be divided into ID2-GFP expressing cells and non-expressing cells (Carotta et al., 2011). When these populations were subjected to *in vitro* differentiation conditions that favor B, T, or NK cell development, only ID2 expressing NKP cells contained NK cell committed progenitor cells. More recently, Fathman et al. (2011) identified similar progenitors to our pre-pro NK and ID2^+^NKP using the surface marker CD244 and CD27. Lin^-^CD27^+^CD244^+^CD127^+^CD117^low^ bone marrow progenitors were divided into CD135^+^CD122^-^CLP, CD135^-^CD122^-^ pre NK, and CD135^-^CD122^+^ NKP, with the latter named rNKP. *In vivo* and *in vitro* assays revealed that, like pre-pro NK and ID2^+^ NKP, pre NK and rNKP were committed to the NK cell lineage (Fathman et al., 2011). Since pre-pro NK and pre NK cells show high similarity, as do Id2^+^NKP and rNKP, it is currently unclear if these populations are identical or if they constitute distinct stages of NK cell commitment. In light of the recent findings, a differentiation model is proposed in which CLPs give rise to pre-pro NK cells, then ID2^+^NKP which in turn give rise to immature and ultimately mature NK cells and propose to unify the nomenclature of early NK development into pre-pro NK cells and rNKP (**Figure 1**).

An open question in the field of innate immunity to date is the existence of a hypothetical progenitor, the common innate lymphocyte progenitor (CILP). CILP are proposed to be the bone marrow precursor of cNK, RORγt-dependent ILCs and Gata3-dependent ILC (also known as ILC2 or nuocytes). To date, pre-pro NK cells have not been tested for ILC or ILC2 potential and it will be of interest if the pre-pro NK population is indeed the earliest NKP or represent the proposed CILP.

#### Transcriptional regulation of the commitment of NK cell from lymphoid progenitors

Several key transcription factors have been identified that regulate the commitment of lymphoid progenitors into T or B cells (Nutt and Kee, 2007). Surprisingly, only a few transcription factors so far have been discovered to regulate the earliest steps that commit lymphoid progenitors to the NK cell lineage. Even less understood is how these factors implement the NK cell-specific transcriptional network. Due to the heterogeneous nature of the initially discovered lin^-^CD122^+^CD49b^-^NK1.1^-^ NKP, most NK cell commitment factors are described to act after the NKP cell stage (Yokota et al., 1999; Boos et al., 2007; Gascoyne et al., 2009; Kamizono et al., 2009). It is therefore possible that the described phenotype has been masked by the impurity of NKP population and re-investigation of the phenotype of mice deficient in these factors using the developmental scheme outlined in **Figure 1** is warranted. Support for this hypothesis comes from a recent study that reinvestigated early NK cell development in Ets-1^-/-^ mice which indeed found that Ets-1 deficiency already affects the formation of the earliest NK cells precursors (Ramirez et al., 2012). Here we review the transcription factors that have been found to function in the regulation of early NK cell development.

***Inhibitor of DNA binding 2*** Inhibitor of DNA binding 2 is expressed in many different hematopoietic tissues (Jackson et al., 2011). Mice deficient in *Id2* lack peripheral lymphoid tissues such as Peyer’s patch and lymph nodes, most cNK cells, dendritic cells, and T cells (Yokota et al., 1999; Boos et al., 2007). ID proteins lack a DNA-binding region and are though to function solely by dimerization with other transcription factors mediating inhibition in a dominant negative manner. Regulation of lineage development is mediated by the interaction of basic helix-loop-proteins of the E-box binding family (E2A, HEB, E2-2) with ID proteins (Quong et al., 2002). Of the four ID family members, *ID2* and *ID3* are the main proteins involved in lymphoid development and control the activity of E-proteins (Quong et al., 2002). Previous work has shown that Id2 is required for cNK cell development, with a strong decrease in bone marrow resident immature NK (iNK) cells. Whether *ID2* acts even earlier and determines NK cell commitment from CLPs is not clear due to the ill defined early NKP as well as the formation of the recently identified pre-pro NK cells in the absence of ID2 has not been analyzed yet (Yokota et al., 1999; Boos et al., 2007). Given the fact that the earliest NK cell precursor, pre-pro NK cells, already express high levels of ID2 (Carotta et al., 2011), determining the formation of the earliest NKP in the absence of ID2 will be of interest. Inhibition of E-protein target genes is required for the propagation of NK cells past this NKP/immature stage (Boos et al., 2007). Furthermore, if E2A is also deleted in Id2^-/-^ NK cells (E2A^-/-^Id2^-/-^) NK cell development in the bone marrow appears to return to normality (Boos et al., 2007). Peripheral mature NK cells are still reduced in E2A^-/-^Id2^-/-^ mice to the level observed in Id2^-/-^ mice suggesting that expression of E-protein target genes contributes to the block in Id2^-/-^ NK cell development in the bone marrow, but Id2 itself is still required to permit normal NK cell maturation (Boos et al., 2007). It is difficult to speculate which E2A regulated genes might be involved in limiting NK cell commitment since our knowledge of E-protein target genes is incomplete.

***Nuclear factor IL-3 (E4BP4)*** Nuclear factor IL-3 (NFIL3), also known as E4-binding protein 4 (E4BP4), belongs to the mammalian basic leucine zipper transcription factor family and functions in a variety of cellular processes such as in the transcriptional control of circadian clock, neural development, and survival to mention a few (Male et al., 2012). Within the hematopoietic system, NFIL3 functions in the macrophage, B, T, NKT, and dendritic cell lineages. Within the NK cell lineage, loss of NFIL3 results in a strong reduction of mature cNK cells (Gascoyne et al., 2009; Kamizono et al., 2009). As for Id2^-/-^ mice, NKP were still present in Nfil3^-/-^ mice. Given the severity of the KO phenotype and because NFIL3 is already expressed in lineage negative bone marrow cells as well as NKP (Gascoyne et al., 2009), re-examination of the effect of NFIL3 within the earliest NK cell populations will be informative on whether NFIL3 is a key factor that induces NK cell commitment or functions at a later stage, potentially downstream of Ets-1 (Ramirez et al., 2012), to implement NK cell fate.

Mechanistically it has been proposed that NFIL3 functions upstream of ID2, as ID2 expression was reduced in Nfil3^-/-^ bone marrow progenitors and enforced expression of ID2 in NFIL3 deficient NK cells could partially rescue NK cell development (Gascoyne et al., 2009). However, it is currently not known if the potential regulation of ID2 by NFIL3 is direct or indirect. Furthermore, loss of NFIL3 expression resulted in only approximately 30% reduction of ID2 expression (Gascoyne et al., 2009). As Id2 heterozygous mice have normal NK cell numbers it remains unclear how important the proposed regulation of Id2 by NFIL3 is for NK cell development (Yokota et al., 1999; Boos et al., 2007).

NFIL3 was also proposed to regulate IL-15 signaling as NFIL3-deficient bone marrow progenitor could not respond to IL-15 stimulation and ectopic NFIL3 expression allowed some NK cell development in the absence of IL15 signaling (Gascoyne et al., 2009). Interestingly, NKT cells, which express NFIL3 and also depend on IL-15 signaling for development and homeostasis, were not found to be affected in NFIL3-deficient mice (Gascoyne et al., 2009; Kamizono et al., 2009), suggesting NFIL3 is one of the key regulators of NK cell development, however, much further work is needed to when and how NFIL3 functions.

***Ikaros (IKZF1)*** Ikaros is the founding member of a small family of Krüppel-type zinc finger transcription factors. Ikaros is expressed as early as in the hematopoietic stem cell and is widely expressed in lymphoid and myeloid progenitor cells (Yoshida et al., 2006). Several Ikaros mutant mouse models have been generated which revealed an essential role of Ikaros in lymphoid development and homeostasis. Ikaros null mice lack all B, T, and NK cells. NK cells are virtually absent in the spleen of Ikaros-deficient mice and Ikaros-deficient progenitors were not able to differentiate into NK cells *in vitro* (Georgopoulos et al., 1992, 1994; Wang et al., 1996; Morgan et al., 1997; Payne et al., 2003). Because Ikaros is expressed in NK cells and Ikaros null mice lack NK cells, it is currently widely postulated that Ikaros is an essential regulator of NK cell commitment (Georgopoulos et al., 1997; Hesslein and Lanier, 2011). However, it cannot be excluded that the block of lymphoid development in Ikaros null mice might be as early as at the CLP stage (Yoshida et al., 2006) as Ikaros regulates cytokine receptors that are important for the generation of CLPs such as CD135 and Ikaros null mice lack or have strongly reduced numbers of B, T, and NK cells. The generation of mice that allow the conditional deletion of Ikaros at or after the CLP stage as well as in committed NK cells would reveal the exact stages in which Ikaros functions in NK cell development.

**FIGURE 2:**
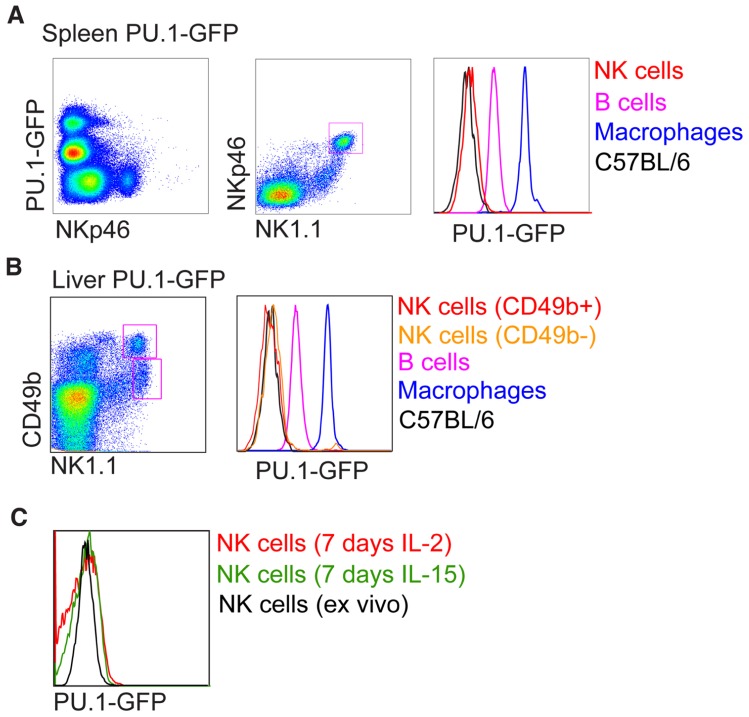
**Analysis of PU.1-GFP expression in NK cells from PU.1 reporter mice**. Intensity of PU.1-GFP levels in **(A)** spleen, **(B)** liver, and **(C)** *in vitro* expanded splenic NK cells were determined by fluorescence-activated cell sorter (FACS) and compared to B cells and macrophages from PU.1-GFP reporter mice or NK cells from C57BL/6 mice.

***PU.1 (Sfp1)*** PU.1 belongs to the Ets transcription factor family. Like Ikaros, PU.1 is expressed at high levels in HSC and continues to be expressed and function in many hematopoietic lineages (Nutt et al., 2005). Pu.1^-/-^ mice die in late gestation or within the first days after birth and lack all lymphocytes as well as granulocytes and macrophages. Conditional deletion of Pu.1 in adult HSC resulted in the absence of lymphoid cells as well as macrophages, while granulocytes were expanded. PU.1 has been shown to have a critical role in the development of CLP in part by regulating receptors essential for proliferation, survival, and differentiation of CLP (Scott et al., 1994, 1997; McKercher et al., 1996; Spain et al., 1999; Carotta et al., 2010b). The development of mature B, T, and NK cells following engraftment of Pu.1^-/-^ fetal liver into alymphoid (Rag2^-/-^gC^-/-^) mice is blocked (Colucci et al., 2001). Because PU.1 protein was detected in the lysates of IL-2 expanded NK cells and it was concluded that PU.1 was required for normal NK cell genesis, although this requirement is less stringent to that of B and T cell development (Colucci et al., 2001). However, re-investigation of PU.1 expression in hematopoietic cells at a single cell level using a PU.1-GFP reporter mouse model revealed no expression of PU.1 in pre-pro NK cells as well as mature NK cells (Nutt et al., 2005; Carotta et al., 2010a). This is supported at the mRNA level (Immgen; www.immgen.org). We repeated the experimental condition of Colucci et al. (2001) by growing sorted NK1.1^+^CD3^-^ NK cells in recombinant IL-2 or IL-15 for 7 days, however, failed to detect any PU.1-GFP expression (**Figure 2**). It is possible that the small population of Pu.1^-/-^ NK cells detected in recipient mice are derived from a subset of PU.1-independent CLPs, or they are the remnants of Pu.1^-/-^ fetal liver NK cells or progeny of Pu.1^-/-^ fetal liver NKPs contained within the graft. While Colucci et al. (2001) did not investigate this possibility, it was recently shown that the E14.5 fetal liver contains a clear population of NKP and NK cells (Tang et al., 2012). E18.5 fetal NKPs readily differentiated into mature NK cells *in vitro* whereas B, T, and myeloid potential are absent (Tang et al., 2012). Lastly on this issue, liver NK1.1^+^CD49b^-^TRAIL^+^ NK cells in Pu.1-GFP^+^ mice were also negative for PU.1 expression (**Figure 2**) and RNA sequencing of CLPs and pre-pro NK cells from wild type bone marrow reveals that PU.1 expression is shut off upon commitment to the NK cell lineage, with pre-pro NK cells and mature NK cells having similarly negligible expression of PU.1 RNA (our unpublished observations). PU.1 has been reported to directly regulate the expression of the cytokine receptors Flt3 and IL7Rα, both of which are expressed on CLPs (DeKoter et al., 2002; Carotta et al., 2010a). Flt3/IL7Rα double deficient mice show defective B, T, and NK cell development (Vosshenrich et al., 2003) and it is thus likely that PU.1 is not a specific regulator of NK cell development but instead a key regulator of CLP development.

***ETS-1*** is the founding member of the Ets transcription factor family. It is expressed in a variety of hematopoietic lineages and Ets1-deficient mice have defects in T, B, and NK cells. However, Ets1^-/-^ mice develop normal numbers of CLPs but do have reduced pre-pro NK, rNKP, and mature NK cells, indicating that ETS-1 is a key regulator of early NK cell development (Barton et al., 1998; Ramirez et al., 2012). A recent study found that by comparing the gene expression of wild type mature and residual Ets-1-deficient NK cells as well as chromatin immune-precipitation studies that ETS-1 potentially regulates early NK cell commitment by directly regulating ID2 expression as well as T-bet, an important transcription factor normal NK cell development (Ramirez et al., 2012). Other potential ETS-1 target genes that might explain the severe block in NK cell development include, Jak3, an essential mediator of IL-15 receptor signaling (Park et al., 1995; Aringer et al., 2003). However, a failure of Ets1-null NKP or iNK cells to respond normally to IL-15 seems unlikely as mature Ets1^-/-^ NK cells appeared if anything hyper-responsive to IL-15 *in vitro* (Ramirez et al., 2012).

***STAT5*** The activation of STAT5 is an early signaling event following IL-15 binding the IL-15 receptor complex. IL-15 binding results in Jak1/Jak3 activation and phosphorylation of STAT5 that is recruited to phosphorylated IL-15 receptors via their SH2-domain (O’Shea and Plenge, 2012). Phospho-STAT5 then form dimers and translocate to the nucleus to initiate transcription of target genes namely involved in cytokine signaling, cell cycle, and survival (Malin et al., 2010; O’Shea and Plenge, 2012). There is no direct evidence that STAT5 activates genes involved in NK cell development such as Id2, Nfil3, Ets1, T-bet despite STAT5-null mice having dramatically reduced NK cell numbers (Nakajima et al., 1997; Imada et al., 1998). STAT5 is suggested to indirectly regulate IL-2Rβ expression (Basham et al., 2008) and most evidence would point to a block in IL-15 signaling as the cause of the NK cell phenotype in Stat5-null mice (Nakajima et al., 1997; Imada et al., 1998). Deletion of Stat5 uniquely in NK cells using STAT5^fl/fl^ mice bred to BAC NCR1-Cre mice resulted in a specific reduction in peripheral NK cells by three- to fivefold compared to non-Cre expressing mice confirming that STAT5 is critical for the maintenance of mature NKp46^+^ NK cells *in vivo* (Eckelhart et al., 2011).

### THYMIC NK CELL DEVELOPMENT

Thymic NK cells differ from cNK cells in several aspects: while cNK development depends on IL-15 and have high cytolytic and cytokine secretion potential, thymic NK development depends on IL-7 signaling, possess weak cytotoxicity but are strong producers of interferon-gamma (IFN-γ), granulocyte–macrophage colony-stimulating factor and tumor necrosis factor (Huntington et al., 2007b). Several obvious differences in the molecular regulation between thymic and cNK cell development exist. Firstly, high expression of E-protein target genes would precede commitment to the NK cell lineage in the thymus as NK cells can be derived from DN thymocytes expressing levels of ID2 and high levels of E2A. This is not predicted to be the case in the bone marrow where increasing expression of ID2 or E-proteins target genes are thought to directly influence lymphoid fate with NK cells development being synonymous with high ID2 and low E-protein target gene expression. Furthermore, thymic NK cells have been proposed to develop independently of ID2 (Boos et al., 2007). Intriguingly, NK cells in periphery of Id2^-/-^ mice also have higher expression of CD127 and lower levels of CD11b and CD43, a phenotype reminiscent of thymic NK cells (Vosshenrich et al., 2006; Boos et al., 2007).

### GATA3

GATA3 expression was shown to be required for thymic NK cell development using transfers of Gata-3 deficient fetal livers into Rag2^-/-^ mice (Vosshenrich et al., 2006). GATA3 is highly expressed in DN2/DN3 thymocytes and required for T cell progression past this stage (Ting et al., 1996; Hendriks et al., 1999; Hosoya et al., 2009). Since thymic NK cells can be derived from DN1/DN2 thymocytes (Li et al., 2010; Vargas et al., 2011) the role for GATA3 in thymic NK cell generation could reflect the importance of GATA3 in DN thymocyte homeostasis. In contrast, cNK cells develop normally in the absence of GATA3 with a phenotype noted in IFN-γ production and liver homing (Samson et al., 2003).

### HUMAN THYMIC NK CELLS

IL-15 promotes the differentiation or expands committed progenitors from human CD34^+^CD1a^-^ thymocytes in FTOCs (Schotte et al., 2010) and while the phenotype of this progenitor is shared by conventional HSCs, Notch signaling is present in this system, confirming murine studies that Notch is not detrimental to NK lineage commitment (Carotta et al., 2006). Thymic CD34^+^CD1a^-^ cells express negligible levels of ID2 compared to thymic NK cells and retroviral overexpression of ID2 greatly promoted NK cell development in FTOCs at the expense of T cells. NK cells were not generated when overexpressing ID2 in thymic CD34^+^CD1a^-^ cells were cultured on OP9-DL1 with IL-7 and Flt-3L indicating IL-15 is essential for this process and that OP9 cells fail to deliver this. While CD34^+^CD1a^-^ thymocytes express higher levels of ID2 than ID3, overexpression of ID3 in fetal liver progenitors yields a similar phenotype with a T cell development block in favor of NK cells (Heemskerk et al., 1997). The difference in requirement for ID2 between human and mouse thymic NK cells may also explain their phenotypic differences with mouse thymic NK cells unanimously expressing CD127 (Vosshenrich et al., 2006), whereas only a minor fraction of NK cells in the human thymus expresses CD127 (Schotte et al., 2010).

### FETAL LIVER NK CELL DEVELOPMENT

Natural killer cells develop and reside in the fetal liver before birth in both man and mouse although their role at this early stage of development is unclear (Phillips et al., 1992; Takeda et al., 2005; Tang et al., 2012). Murine fetal NK cells are present from E14 (Tang et al., 2012) and are predominantly NK1.1^+^CD49b^-^TRAIL^+^Mac-1^-^Ly49^-^ and not dependent on Eomes for their development, which contrasts adult cNK cells being NK1.1^+^CD49b^-^TRAIL^+^Mac-1^+^Ly49^+^ and Eomes-dependent (Gordon et al., 2012). NK1.1^+^CD49b^-^TRAIL^+^Mac-1^-^Ly49^-^ NK cells are maintained in the liver into adulthood, however, the size of this population reduces over time as cNK cells develop and represent the majority of the NK cell compartment in the liver of adult mice (Takeda et al., 2005; Huntington et al., 2007a). Evidence suggests that cNK cells can be derived from liver NK1.1^+^CD49b^-^TRAIL^+^Mac-1^-^Ly49^-^ NK cells following adoptive transfer (Takeda et al., 2005; Gordon et al., 2012), however, the contribution of the fetal liver NK cells versus bone marrow pre-pro NK cells to the cNK cell pool *in vivo* remains to be examined.

The transcriptional requirement for fetal NK cell development is also an area of uncertainty, although this population is dependent on γC signaling, because we do not detect this population (NK1.1^+^CD49b^-^TRAIL^+^Mac-1^-^Ly49^-^) in Rag2^-/-^γC^-/-^ mice (Huntington et al., 2007a). Differential transcription factor dependence of fetal versus cNK cell development could also influence experiments where fetal liver transfers are used as a source of HSCs. Indeed in the case of PU.1, we do not know whether Pu.1^-/-^ E14.5 fetal livers contain NK cells and NKPs but the possibility cannot be ruled out. The layered immune system theory also supports the idea that fetal and adult immune cells of the same lineage have different gene regulation pathways that dictate functional responses to pathogens such as tolerogenic versus immunogenic in fetal and adult cells, respectively (Mold and McCune, 2011, 2012). When transferred into Rag2^-/-^γC^-/-^ mice, NK1.1^+^CD49b^-^TRAIL^+^Mac-1^-^Ly49^-^ NK cells gave rise to NK cells resembling cNK cells (Gordon et al., 2012) and since we know that immature and mature NK cells undergo homeostatic expansion in Rag2^-/-^γC^-/-^ mice (Huntington et al., 2007a), any Pu.1^-/-^ fetal liver derived NKP or NK cells could give rise to the small number of NK cells reported in the Rag2^-/-^γC^-/-^ mice by Colucci et al. (2001) without having passed through the convention bone marrow pathway where PU.1 deletion appears to block CLP development.

## CONCLUDING REMARKS AND OPEN QUESTIONS

Several transcription factors have been identified to regulate the development of lymphoid progenitors into NK cells. As indicated above, for several of them it is still unclear at what developmental stage they act. Re-examination of earliest steps of NK cell development in these KO mice will be informative in which order these transcription factors work and how they influence NK lineage commitment and repress alternative lineage fates. The future determination of target genes of these factors will be of immense importance for our understanding of the transcriptional network that regulates NK cell commitment. Most of these transcription factors are not only expressed during the earliest steps of NK cell development but continue to be expressed during NK cell development. It will also be of interest to determine if these factors are also important regulators of NK cell function.

## Conflict of Interest Statement

The authors declare that the research was conducted in the absence of any commercial or financial relationships that could be construed as a potential conflict of interest.
